# TIM proteins and microRNAs: distinct impact and promising interactions on transplantation immunity

**DOI:** 10.3389/fimmu.2024.1500228

**Published:** 2024-11-22

**Authors:** Jialing Tao, Xiaoxuan Shen, Haiqing Qian, Qing Ding, Lihong Wang

**Affiliations:** ^1^ Translational Medical Innovation Center, Zhangjiagang TCM Hospital Affiliated to Nanjing University of Chinese Medicine, Zhangjiagang, Jiangsu, China; ^2^ Department of Endocrinology, Zhangjiagang TCM Hospital Affiliated to Nanjing University of Chinese Medicine, Zhangjiagang, Jiangsu, China; ^3^ Department of Reproduction, Zhangjiagang TCM Hospital Affiliated to Nanjing University of Chinese Medicine, Jiangsu, Zhangjiagang, China; ^4^ Department of Surgery, Thomas E. Starzl Transplantation Institute, University of Pittsburgh School of Medicine, Pittsburgh, PA, United States

**Keywords:** T cell immunoglobulin and mucin domain, microRNAs, transplantation, allograft rejection, allograft tolerance

## Abstract

Achieving sustained activity and tolerance in of allogeneic grafts after post-transplantation remains a substantial challenge. The response of the immune system to “non-self” MHC-antigenic peptides initiates a crucial phase, wherein blocking positive co-stimulatory signals becomes imperative to ensure graft survival and tolerance. MicroRNAs (miRNAs) inhibit mRNA translation or promote mRNA degradation by complementary binding of mRNA seed sequences, which ultimately affects protein synthesis. These miRNAs exhibit substantial promise as diagnostic, prognostic, and therapeutic candidates for within the realm of solid organ transplantations. Current research has highlighted three members of the T cell immunoglobulin and mucin domain (TIM) family as a novel therapeutic avenue in transplantation medicine and alloimmunization. The interplay between miRNAs and TIM proteins has been extensively explored in viral infections, inflammatory responses, and post-transplantation ischemia-reperfusion injuries. This review aims to elucidate the distinct roles of miRNAs and TIM in transplantation immunity and delineate their interdependent relationships in terms of targeted regulation. Specifically, this investigation sought seeks to uncover the potential of miRNA interaction with TIM, aiming to induce immune tolerance and bolster allograft survival after transplantation. This innovative strategy holds substantial promise in for the future of transplantation science and practice.

## 1 Introduction

Allogeneic transplantation is the primary treatment for patients with end-stage diseases and severe trauma. Imbalances in the activation and suppression of the immune system, systemic dysfunction of the transplanted organ, and infections all contribute to the failure of allogeneic transplantations (1–3). In many cases, autologous transplantation is not feasible due to physiological restrictions (4, 5). Consequently, allogeneic transplantation remains the only viable solution in such scenarios. However, graft rejection remains a major obstacle leading to graft loss (6).

The T cell immunoglobulin and mucin domain (*TIM*) gene family comprises a series of genes encoding type 1 glycoprotein-like structural domains expressed on cell membranes that crucially regulate immune responses (7). Members of the *TIM* gene family, such as TIM-1, TIM-3, and TIM-4, exhibit structural characteristics that are conserved in both mice and humans (8). Initially identified as a susceptibility gene for asthma and allergy, TIM-1 is preferentially expressed on Th2 cells and linked to atopic and autoimmune diseases (9). TIM-3 is expressed on innate and adaptive immune cells, including mast cells, dendritic cells (DCs), macrophages, and Th1 and Tc1 cells, and acts as an inhibitory receptor that promotes Th1 apoptosis and reduces the production of inflammatory factors (10–12). TIM-4 is solely expressed on the surface of antigen-presenting cells (APCs), facilitating phagocytosis of apoptotic cells and modulating T cell responses (7, 13). Ongoing research underscores the extensive role of TIM proteins in immune tolerance and transplant rejection (14–16).

MicroRNAs (miRNAs), single-stranded RNAs approximately 22 nucleotides long, selectively and specifically regulate post-transcriptional gene expression (17). Recently, miRNAs have demonstrated specific and impactful biological effects, serving to establish immune tolerance following solid organ transplantation (18). Thus, miRNAs exhibit potential as diagnostic, predictive, and therapeutic markers for allograft rejection (19).

Both miRNAs and TIM proteins have wide applications in immune tolerance induction and transplantation (20). The interaction between miRNAs and TIM proteins in cancer therapy has been extensively studied (21, 22) (**Table 1**). However, their effects on allograft rejection models remain unclear. Thus, this review aims to discuss recent advancements in understanding the TIM–miRNA network and explore its potential applications in solid organ transplantation and immune tolerance.

**Table 1:** TIM–miRNA interactions in diseases.

TIM	miRNA	Effect	Reference
TIM-1	miR-133a	Targeted regulation of glioblastoma cell proliferation, migration, and infiltration.	(21)
	miR-142	Alteration of endothelial cell permeability.	(23)
TIM-3	miR-330	Inhibition of NLRP3 inflammasome-mediated myocardial ischemia-reperfusion injury. Insulin resistance downregulated by enhancing M2 macrophage polarization. Mediation of anti-tumor immunity in AML.	(24–26)
	miR-125a-3p	Negative effect on AML progression.	(27)
	miR-498	Potential approaches for the treatment of AML.	(22)
	miR-18b	Improved pre-eclampsia by promoting trophoblast proliferation and migration.	(28)
	miR-34a	Modulates the degree of malignancy in AML	(29)
	miR-155	Regulation of CD8 T cell apoptosis and improved immunotherapy efficacy in hepatocellular carcinoma. Blocks macrophage transformation to prevent the development of atherosclerosis. Predicts colorectal cancer progression by targeting macrophage polarization. Accelerates cervical cancer progression by modifying the macrophage microenvironment.	(30–33)
	miR-455-5p	Predicts clinical regression in patients with skull base chordoma.	(34)
	miR-545-5p	Modulates the anti-tumor activity of CD8 T cells	(35)
	miR-149-3p	Anti-tumor immunity in breast cancer by reversing CD8 T cell depletion.	(36)
	miR-133a	A future therapeutic target in AML.	(37)
	miR-146a	A predictor of cellular immune failure following HIV infection.	(38)
TIM-4	miR-202	Acceleration of EC cell migration and invasion by targeting the miR-202–TIM-4 axis.	(39)

AML, acute myeloid leukemia; IL, interleukin; miRNA, microRNA; TIM, T cell immunoglobulin and mucin domain.

## 2 *TIM* gene family

**Figure 1:**
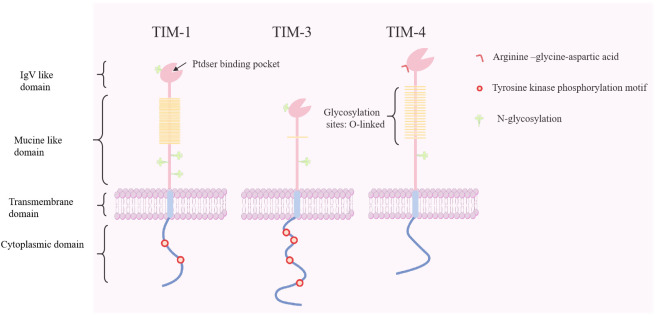
Molecular structure of human T cell immunoglobulin and mucin proteins (TIM-1, -3, and -4). The *TIM* genes encode type I membrane proteins that contain an Ig V-like domain, an O-linked glycosylated mucin domain, a transmembrane domain, and a cytoplasmic domain with tyrosine-phosphorylated motifs. TIM-4 contains RGD motifs that can interact with integrins and participate in intercellular adhesion. Ptdser, phosphatidylserine; RGD, arginine−glycine−aspartic acid.

The *TIM* genes are located on mouse chromosome 11B1.1 and human chromosome 5q33.5, which are regions associated with various atopic/autoimmune diseases such as asthma and allergies (40). The TIM family comprises eight murine members (four coding genes, TIM-1–TIM-4, and four noncoding genes, TIM-5–TIM-8) and three human members (TIM-1, TIM-3, and TIM-4) (41). TIM proteins share a similar structure, encompassing an immunoglobulin domain, mucin-like domain, transmembrane region, and cytoplasmic domain containing tyrosine-phosphorylated motifs (except for TIM-4) (12) (**Figure 1**). Based on gene sequence similarity, murine TIM-2 shares structural and functional similarities with murine TIM-1, and is considered a direct homolog of human TIM-1 (42).

### 2.1 Functional characteristics of TIM-1

Initially identified as the hepatitis A virus receptor (*HAVCR1*) and later as a human kidney injury molecule, TIM-1 is found on B cells, DCs, mast cells, and invariant natural killer T (iNKT) cells, playing a crucial role in immune activation (43–45). As a potential co-stimulatory molecule, it is well established that TIM-1 exerts immune effects by maintaining Breg suppression and stimulating effector T cell activity and homeostasis (8, 46). The diverse biological roles of TIM-1 open up new avenues for the treatment of autoimmune diseases, viral infections and tumors (47–49). Previous studies have suggested the potentially diverse roles of TIM-1 in inducing immune tolerance in transplantation.

### 2.2 Role of TIM-1 in transplantation

Recent studies have highlighted the pivotal role of TIM-1 in preventing and mitigating allograft rejection. The agonistic TIM-1-specific mAb 3B3 disrupts mouse allograft tolerance by interacting with effector T cells and Tregs (50). Additionally, TIM-1 not only serves as a surface marker but also as a crucial surface molecule that induces and maintains regulatory B cell (Breg) function in mice (51, 52). In a model of islet transplantation, anti-CD45RB and anti-TIM-1 (RMT1-10) antibodies increased interleukin (IL)-10 expression in TIM-1^+^ Bregs and antigen-specific transplantation tolerance (52, 53). This combined antibody therapy relies on TIM-1 expression, IL-10-producing Bregs, and Tregs (54). Altered IL-10 levels and accelerated allograft rejection have been observed in TIM-1 knockout and mutant mice (46). Recent findings indicate that the inhibitory function of ex vivo expansion of human B cells partly relies on TIM-1, which maintains long-term regulatory function and human allogeneic skin graft survival by positively regulating STAT3 phosphorylation (55). The TIM-1 signaling pathway is not only targeted after allogeneic transplantation, but also as a new therapeutic strategy to improve post-transplant complications (56).

### 2.3 Functional characteristics of TIM-3

TIM-3 serves as a suppressor molecule involved in T cell activation and is a marker of T cell depletion in tumors and chronic viral infections (57). Subsequently, TIM-3 was found to accelerate tumor progression and support maternal-fetal tolerance (58, 59). Galectin-9 (Gal-9), the first ligand identified for TIM-3, eliminates interferon γ-producing Th1 cells, thereby reducing the severity and mortality of experimental autoimmune encephalomyelitis (60) (**Table 2**). TIM-3 interacts with different ligands and mediates various immune responses, making it a promising target for immunotherapy.

**Table 2:** Expression and function of TIM proteins.

TIM	Ligand	Expression	Function	Reference
TIM-1	TIM-4	Activated CD4^+^ T cells	Inhibition of interactions that modulate Th1/Th2 cytokine balance and attenuate renal IRI. Modulation of helper T cell activation and proliferation. Amelioration of Behcet’s disease-like symptoms. Suppression of interactions inhibiting DC maturation and CD4^+^ T cell proliferation, thereby inducing immune tolerance.	(61) (62, 63) (64)
		Th2	Exacerbates allergies/asthma.	(65)
	Ptdser	T cells	Enhanced viral cell attachment and infection.	(66, 67)
		iNKT cells	Inhibition of IL-17A production by γδ T cells via PD-1/PD-L1 signaling.	(68)
	P-/E-/S-selectin	Th1/Th17 cells	Binding, rolling, and accumulation of Th1 and Th17 cells in the local microenvironment during inflammatory disease.	(69)
	HAV	Proximal tubule cells	A marker of renal injury. Mediate fatty acid uptake; exacerbates inflammation and renal fibrosis, and accelerates the progression of diabetic nephropathy.	(70)
	LMIR5/CD300b	Epithelial tubular cells	Promote neutrophil recruitment to kidneys with IRI, thereby facilitating renal injury.	(71)
TIM-3	Gal-9	Th1, Tc1, and NK cells	Negative regulation of Th1 and CD8 T cell responses, promotion of Treg development to rescue inflammatory injuries after transplantation, and induction of immune tolerance. Modifies NK function, balances the Th1/Th2 ratio, and promotes maternal and fetal tolerance to prevent abortion.	(72) (73)
		T cells	PD-1 attenuates Gal-9/TIM-3-induced T cell apoptosis by binding to Gal-9, providing a novel target for anti-tumor immunity.	(74)
		Macrophages	Prevent macrophage M2 polarization by blocking Gal-9/TIM-3 signaling in *PTEN*-deficient gliomas, thereby attenuating glioma progression.	(75)
		NK	Drives NK cell dysfunction and immune escape in AML.	(76, 77)
	HMGB1	CD8 T cells/DCs	Accelerate viral infection by limiting effector T cell activation and amplification	(78)
		T cells	Promote AML progression.	(79)
			Block NF-ΚB activation, modulates immunosuppression, and increases mortality in sepsis.	(80)
	CEACAM1	T cells	T cell depletion and inhibited signaling	(57)
		CD4 T cells	Reduces= stress-induced tissue damage, inhibits Kupffer cell activation, and improves outcomes in liver transplantation.	(81)
		CD8 T cells	Regulation of premature restimulation-induced cell death of effector CD8 T cells and stabilization of T cell populations.	(82)
		T/NK/B cells	A potential target for anti-tumor immunity/autoimmune diseases.	(83–85)
	Ptdser	NK/CTL cells	Influence cell toxicity and mediates immune escape from malignant tumors.	(86, 87)
TIM-4	TIM-1	PMBCs	Possible involvement in the pathogenesis of systemic lupus erythematosus.	(88)
		B cells	Promote tumor and graft rejection.	(89)
			Promote Th2 proliferation and exacerbates allergic rhinitis.	(90)
	Ptdser	Macrophages	Facilitate viral entry into target cells	(91, 92)
			Scavenges apoptotic cells to avoid autoimmunity.	(93)

AML, acute myeloid leukemia; AHR, airway hyperreactivity; CEACAM1, carcinoembryonic antigen cell adhesion molecule 1; CTL, cytotoxic T cell; DC, dendritic cell; HAV, hepatitis A virus; Gal-9, galectin-9; HMGB1, high-mobility group protein B1; IL, interleukin; iNKT, invariant natural killer T; IRI, ischemia-reperfusion injury; NK, natural killer; PBMC, peripheral blood mononuclear cell; PD-1, programmed cell death protein 1; Ptdser, phosphatidylserine; TIM, T cell immunoglobulin and mucin domain.

### 2.4 Role of TIM-3 in transplantation

Initially considered as a marker for terminally differentiated effector T cells, TIM-3 has been found to influence Treg acquisition and function, providing new insights into the mechanisms of transplant rejection (94). The natural TIM-3 ligand Gal-9 limits Th1 activation, thereby protecting specific Treg responses and attenuating allograft rejection (95). When allograft rejection occurs, increased expression of TIM-3 on the recipient’s NK cells stimulates IFN-γ production through interaction with Gal-9 (96). Therefore, high serum levels of soluble TIM-3 and sGal-9 serve as prospective biomarkers for diagnosing and predicting renal transplant dysfunction (97, 98). Additionally, hepatocytic Gal-9 signaling via TIM-3^+^CD4^+^ T cells mitigate ischemia-reperfusion injury (IRI) during orthotopic liver transplantation in recipient mice (72). TIM-3^+^CD4^+^ and TIM-3^+^CD8^+^ T cells in allogeneic transplantation models exhibit a depleted dysfunctional phenotype owing to continuous stimulation by allogeneic antigens (99). This early induction and establishment of T cell dysfunction ultimately mediate and maintain the phenotypic and functional characteristics of self-tolerance or exhaustion (100). Moreover, inhibitory receptors such as TIM-3 and PD-1 ensure that Treg are depleted after graft rejection to prevent microbial and tumor unresponsiveness and to balance immunomodulatory functions (16). High pretransplant T-cell expression of PD-1 and Tim-3 co-suppressor receptors correlated positively with the incidence of posttransplant infection (101). Clinical studies have shown that elevated CEACAM1 levels are associated with a favorable outcome in orthotopic liver transplantation. Recent evidence confirms that T cell CEACAM1 - TIM-3 crosstalk inhibits Kupffer cell NF-ΚB phosphorylation, attenuates post-transplant liver injury and promotes T cell homeostasis (81). Overall, TIM-3 has shown potential applications in transplantation, but more thorough mechanisms of action need to be explored.

### 2.5 Functional characteristics of TIM-4

Traditionally known to be primarily expressed on the surface of APCs, including macrophages, mature DCs, B1 cells, and iNKT cells, recent studies have also identified TIM-4 expression in fibroblasts (13, 102). This diverse expression profile suggests potential multifaceted roles of TIM-4 in immune regulation and cellular interactions. Structurally, despite the lack of a cytoplasmic tail for intracellular signaling, the TIM-4 extracellular IgV domain contains arginine-glycine-aspartate (RGD) motifs, which predominantly facilitates APC-T cell adhesion (7, 103).

Initial studies have suggested that TIM-4 acts as a natural ligand for TIM-1, contributing to helper T cell proliferation and favoring Th2 immune responses (104). However, further investigations have revealed the nuanced effects of TIM-4 on T cell responses. Depending on the concentration of TIM-4 stimulation and the state of T cell activation, TIM-4 has contrasting effects on T cell proliferation (62, 105). These findings suggest that the influence of TIM-4 on T cells may involve receptors other than the known TIM-1 receptor, especially during the initial T cell surface expression.

As a phosphatidylserine receptor, TIM-4 contributes to the creation of an environment of immune tolerance by clearing apoptotic cells and debris, simultaneously suggesting potential risks associated with infection and tumorigenesis (91, 106). Overall, the function of TIM-4 as a potent co-stimulatory signal in APCs revealed its diverse and context-dependent biological activities. Its precise biological effects seem to be closely linked to the type of ligands it interacts with and the specific sites of T cell activation. Understanding the intricate interactions of TIM-4 with various receptors and their dual roles in immune tolerance and potential pathogenic processes remains an area of active research in immunology.

### 2.6 Role of TIM-4 in transplantation

Few studies have investigated TIM-4 in the context of transplantation. Researchers have focused on understanding TIM-4 expression in specific immune cells, particularly macrophages and DCs, as these cells play crucial roles in the modulation of TIM-4 to promote tolerance in human transplantation (107, 108).

Prior to 2010, studies exploring the direct relationship between TIM-4 and transplantation immunity were lacking. However, in 2010, Uchida et al. hypothesized that blocking the TIM-1–TIM-4 signaling pathway might alleviate hepatic IRI. The proposed intervention presented a novel approach aimed at extending the survival and success of transplanted organs (109). In the following year, Rong et al. provided initial evidence supporting this hypothesis by demonstrating that disrupting the TIM-1–TIM-4 pathway could inhibit CD4 T cell activation. This inhibition protected renal function and reduced local leukocyte recruitment and activation, offering a promising novel target for the treatment of acute kidney injury (61).

Subsequent studies further reinforced these initial findings, consistently showing that blocking TIM-4 signaling conferred protection against hepatic IRI. Notably, these studies highlight the significance of TIM-4-mediated phagocytosis, which is involved in activating the innate immune system and represents a crucial aspect of this process (110, 111). Indeed, these studies underscore the potential therapeutic implications of targeting TIM-4 in mitigating transplantation-related complications, and hold promise for developing novel strategies to enhance the success of organ transplantation.

Macrophages, particularly tissue-resident macrophages such as CD169^+^ macrophages, play a critical role in modulating immune responses and influencing transplant outcomes. For instance, genetic ablation of TIM-4 in CD169^+^ tissue-resident macrophages improve their survival. However, this alteration does not seem to affect the effective stimulation of Treg production or promote the prolonged survival of cardiac allografts (15).

Kupffer cells (KCs), the dominant macrophages in the liver, have been identified as critical mediators of tolerance following liver transplantation. KCs promote tolerance through mechanisms involving upregulation of FasL-induced apoptosis and cytokine secretion in T cells (112, 113). Disrupting TIM-4 signaling in KCs in combination with transforming growth factor (TGF)-Β treatment significantly induces the transformation of inducible Tregs and ameliorates acute rejection after liver transplantation. This effect occurs via inhibition of the IL-4–STAT6–Gata3 signaling pathway, thereby modulating immune responses and improving tolerance induction (114).

However, studies on mice with congenital TIM-4 deficiency have reported an autoimmune response due to nonspecific immune activation. This is because of defects in the ability to eliminate apoptotic cells, suggesting a crucial role for TIM-4 in maintaining immune homeostasis and preventing autoimmunity (115). Moreover, DCs, which are highly specialized APCs, are key players in the induction of inflammation and immune tolerance (116). In a skin transplantation model, disruption of TIM-4 co-stimulatory signaling on DCs enhanced the transfer of naïve CD4 cells to inducible Tregs, while limiting the transfer of IL-4/STAT-6 signaling. This modulation attenuates the Th2 response and effectively prolongs graft survival, highlighting the potential of targeting TIM-4 on DCs to modulate immune responses in transplantation scenarios (117).

Collectively, these findings emphasize the intricate role of TIM-4 in regulating immune responses involving macrophages, KCs, and DCs in transplantation scenarios, suggesting its potential as a target for therapeutic interventions to modulate immune tolerance and improve graft survival.

### 2.7 TIM proteins as phosphatidylserine receptors

**Figure 2:**
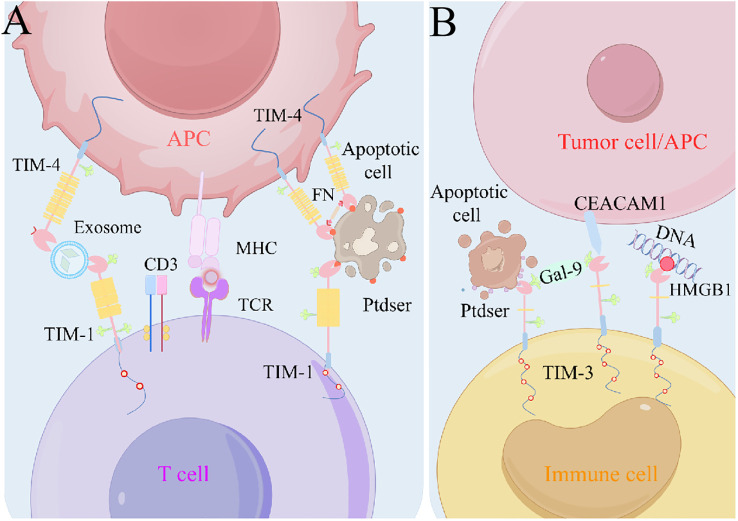
Models of TIM-ligand interactions. **(A)** TIM-1 can interact with Ptdser on the surface of apoptotic cells, or TIM-1 and TIM-4 interact via exosome bridging. TIM-4 is used as a bolus molecule to immobilize apoptotic cells near phagocytes to initiate efferocytosis. **(B)** Gal-9 can promote TIM-3 oligomerization and thus the interaction with other TIM-3 ligands, such as CEACAM1–TIM-3. Ptdser released from apoptotic cells can bind the FG-CC′ cleavage site of TIM-3. In addition, TIM-3 can bind HMGB1 and thus inhibit nucleic acid-mediated anti-tumor immunity. APC, antigen-presenting cell; CEACAM1, carcinoembryonic antigen cell adhesion molecule 1; Gal-9, galectin-9; HMGB1, high-mobility group protein B1; MHC, major histocompatibility complex; Ptdser, phosphatidylserine; TCR, T cell receptor; TIM, T cell immunoglobulin and mucin domain.

Structurally, TIM proteins create a cavity with a distinctive “pocket” structure in the immunoglobulin variable region, securely binding to phosphatidylserine (93, 118) (**Figure 2**). During apoptosis, phosphatidylserine exposure to the plasma membrane triggers phagocytosis, which is essential for tissue homeostasis and immune regulation (119, 120). TIM-1 signaling by T and iNKT cells prevents recipient survival by inhibiting acute graft-versus-host disease after hematopoietic cell transplantation (20). TIM-1-expressing renal epithelial cells aid in phagocytosis of damaged cells, thereby limiting inflammation (121, 122). In addition to its role in phagocytosis, TIM-3 utilizes functional antibodies with phosphatidylserine to enhance T cell activation and anti-tumor activity (123). TIM-4, as a surface receptor, indirectly modulates inflammation and tumor progression through immune cell clearance (43, 106).

## 3 Expression and functions of miRNAs

### 3.1 Biogenesis of miRNAs

**Figure 3:**
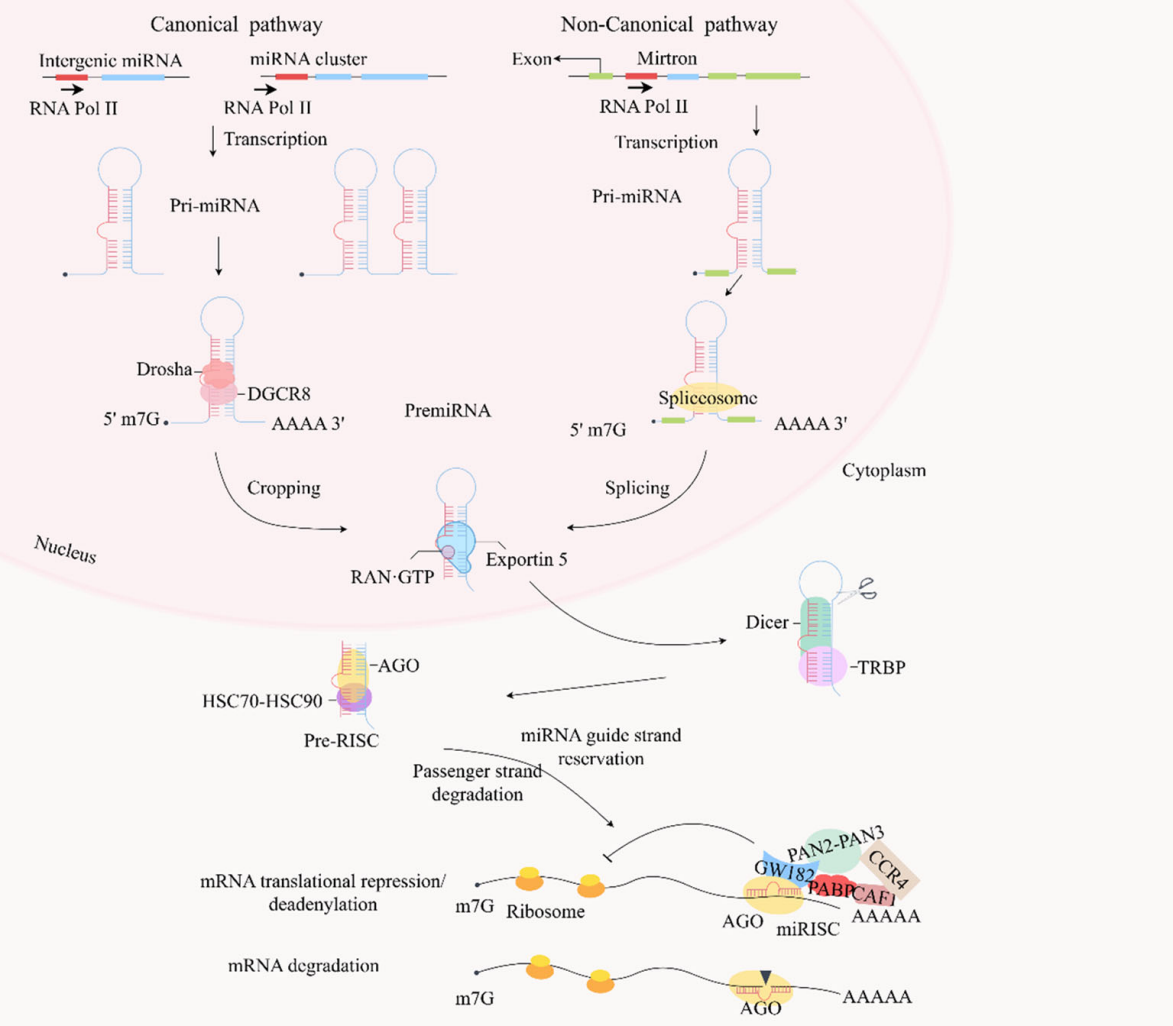
In the canonical pathway, typical miRNA genes are encoded by introns in the transcript, generating single or multiple cis-transcripts, but some miRNAs are encoded by exonic regions. miRNAs in the same cluster are co-transcribed and undergo additional post-transcriptional regulation. Most miRNAs generate primary transcription products (pri-miRNAs) in response to RNA polymerase II, which have the original hairpin structure of the embedded miRNA sequence. The primary precursor (pri-miRNA) is cleaved by the microprocessor complex (including Drosha and DGCR8) into a stem-loop structure of about 70 nucleotides called pre-miRNA. Drosha is an endonuclease responsible for processing and cropping the pri-miRNA, whereas DGCR8 is a protein that binds the pri-miRNA to Drosha. Furthermore, some pre-miRNAs are produced in the nucleus in very short introns (mirtrons) by splicing and debranching without Drosha/DGCR8 processing. The pre-miRNAs are then exported to the cytoplasm via Exportin 5 and RAN-GTP. Dicer in the cytoplasm cleaves the pre-miRNA by TRBP-assisted cleavage of the pre-miRNA, releasing a dsRNA of about 20 bp. The dsRNA is then loaded onto the AGO protein and the HSC70-HSC90 complex. The passenger strand is degraded, and the guide strand is retained in the AGO protein, ultimately forming a RISC. This RISC prevents the initiation of translation by inhibiting ribosome elongation and facilitates de-adenylation of poly(A) by recruiting GW182, PABP, CCR4-CAF1, and PAN2-PAN3 to promote mRNA attenuation. These mRNAs are cleaved and degraded when the RISC can target mRNAs that are nearly fully complementary. dsRNA, double-stranded RNA; miRNA, microRNA; RISC, RNA-induced silencing complex; TRBP, TAR RNA-binding protein.

miRNAs are a class of small noncoding RNAs present in animals, plants, and some viruses that play a crucial regulatory role in transcription by either cleaving target mRNAs or inhibiting their translation (124). The gene sequences encoding miRNAs are arranged differently within the genome. Some miRNAs are organized as mono-cis-parallels with autonomous promoters, whereas others are arranged in multi-cis-parallels, sharing a common promoter and being transcribed into multiple miRNA clusters (125, 126). In certain cases, miRNA genes are located within the exons (**Figure 3**). RNA polymerase II is typically responsible for miRNA transcription. This process generates primary precursors known as pri-miRNAs, which adopt a typical hairpin structure and contain a 5′- and a 3′-polyadenylated tail. Subsequently, pri-miRNA undergoes precise cleavage in the nucleus by Drosha and DiGeorge Syndrome Critical Region 8(DGCR8), a nucleic acid endonuclease of the RNase III family, producing pre-miRNAs with stem-loop structures (127).

The pre-miRNA, approximately 70 nucleotides in length, is formed by the Drosha enzyme and exported from the nucleus to the cytoplasm via Exportin 5. In the cytoplasm, it is further processed by Dicer/TAR RNA-binding protein (TRBP)/AGO into double-stranded RNA (dsRNA) consisting of a guide strand and a passenger strand. The guide strand, typically around 22 nucleotides long, enters the miRNA-induced silencing complex (RISC), leading to translational repression or degradation of the target mRNA, whereas the passenger strand is released and subsequently degraded (124, 128, 129). More recently, it was discovered that miRNA biogenesis can occur independently of the conventional Drosha–DGCR8 pathway. Some pre-miRNAs are produced in the nucleus in very short introns by splicing and debranching (130).

### 3.2 Clustered miRNAs

Approximately 25% of human miRNA genes are organized into clusters, wherein a single cluster contains two or more miRNA genes (131). Although multiple miRNA primary transcripts are generated from the same gene cluster, differential expression arises because of complex regulatory mechanisms. For instance, the 23a–27a–24-2 cluster, comprising three miRNAs, exhibits dysregulation in specific tumors and leukemias, where sometimes only one or two miRNAs are expressed (132). Conversely, some clustered miRNAs show coordinated expression, with a change in a single miRNA gene within the cluster, triggering a chain reaction that affects the other pri/mature miRNAs (133). Current research supports the idea that miRNAs within the same cluster often target overlapping sets of genes, implying enhanced specificity in targeting and increased interconnectedness within the regulatory network (134). miRNA clusters display homogeneity, multiplicity, and paradoxical functions with respect to the roles of individual miRNAs.

### 3.3 Modes of miRNA regulation

miRNAs serve as fundamental components in RISC, which comprises AGO proteins along with certain cofactors (127). Initially, it was believed that miRNAs exert post-transcriptional control over their targets by regulating processes such as translation elongation, protein degradation, and ribosomal release (135). In mammals, the seed sequence at the 5′ end of the miRNA (nucleotides 2-8) recognizes the 3′ or 5′ UTR of the target mRNA (126). Typically, this recognition involves incomplete base pairing, ultimately leading to cleavage and degradation of the target mRNA. In addition, miRNA-mediated target decay and deadenylation ultimately lead to reduced protein production and fine-tuned gene expression (136).

### 3.4 Role of miRNAs in transplantation

The use of miRNAs as noninvasive biomarkers has shown promising potential for the diagnosis, prognosis, and treatment of various aspects of organ transplantation, particularly liver transplantation (137).

#### 3.4.1 Liver transplantation

Reperfusion injury is a major concern after liver transplantation and a leading cause of graft failure and rejection (138). Serum miR-122 levels have been proposed as independent markers of persistent liver injury and early liver allograft dysfunction (139). Hepatocyte-derived miR-122 triggers M1 polarization of KCs, exacerbating hepatic IRI by modulating specific pathways (140). The early elevation of serum levels of miRNAs, including miR-122, miR-146a, and miR-192, has shown potential as powerful markers for predicting graft injury and acute rejection after liver transplantation, often preceding changes in transaminase levels (141).

miR-155 plays a role in inflammation, immunity, and tumorigenesis in liver disease. Inhibition of miR-155 expression in KCs results in positive outcomes by activating anti-inflammatory pathways, enhancing the survival of liver allografts, and attenuating inflammatory injury and apoptosis after IRI (142, 143). MiRNAs such as miR-155 and miR-181a may also serve as potential noninvasive biomarkers. Pre-transplant miR-155 levels identified patients at low immunological risk, and the combination of miR-181a and miR-155 levels acted as an early and noninvasive biomarker for preventing acute T cell-mediated rejection (TCMR) and subclinical rejection (144).

These findings suggest that specific miRNAs hold promise as reliable and early markers for assessing graft injury, predicting rejection episodes, and monitoring complications, such as HCC recurrence after liver transplantation. Further research and validation studies could enhance their clinical utility for improving patient outcomes and graft survival.

#### 3.4.2 Renal transplantation

The use of miRNAs as diagnostic and prognostic markers in renal transplantation has shown considerable potential for addressing various aspects of graft health, rejection, and long-term dysfunction.

Recent validation studies have highlighted that miRNAs, including miR-142-5p, miR-142-3p, miR-155 and miR-223, have high specificity in biopsy specimens and help predict TCMR in allogeneic kidney transplantation (145). Interestingly, Pierre’s group identified a variety of miRNAs that interact with the TIM gene, including miR-142-3p and miR-142-5p by analyzing miRNA profiles in kidney allograft samples. However, alloimmune injury pathways are often not unique or specific, and miRNAs such as miR-142-3p or miR-155-5p have been associated not only with IFTA but also with acute rejection or TCMR (146).

miR-21 is a crucial marker of chronic renal dysfunction after transplantation. Silencing miR-21 directly activates Notch2, inhibits the development of renal fibrosis and inflammation, and ultimately prevents chronic allograft dysfunction (147). Changes in miR-21 expression levels in plasma, urine, and graft tissue serve as diagnostic markers for identifying renal injury and dysfunction over time (148).

Moreover, miRNAs, including miR-19a, miR-886-5p, miR-126, miR-223, and miR-24, have been validated as independent predictors of HCC recurrence within the Milan criteria after liver transplantation, aiding the prognosis and management of HCC after transplantation (149).

Richard and colleagues conducted an analysis of microRNA expression in peripheral blood mononuclear cells (PBMC) from patients with chronic antibody-mediated rejection (CAMR) and those with stable graft function, revealing a significant upregulation of miR-142-5p in CAMR (150). This finding was validated and analyzed, indicating that miR-142-5p functions not only as a potential biomarker for CAMR but also plays a role in regulating the immune status of patients.

TCMR, treatable without causing graft failure but associated with chronic or progressive renal dysfunction, has been associated with specific miRNA profiles, aiding in the prediction and understanding of this type of rejection (151).

These findings underscore the potential of miRNAs as noninvasive and specific biomarkers for diagnosing rejection types, monitoring graft health, and predicting chronic dysfunction in renal transplantation. Continued research and validation are essential to refine their clinical utility and enhance their role in improving patient outcomes after transplantation.

#### 3.4.3 Heart transplantation

The role of miRNAs in heart transplantation has emerged as a promising avenue for diagnosing graft rejection, understanding immune responses, and improving outcomes. Recent studies have shed light on the specific miRNAs associated with acute cellular rejection (ACR) and ABMR after heart transplantation.

Identified and validated in 2020, miR-181a-5p showed promise as a marker for ACR in heart transplantation (152). Its specificity and high negative predictive value render it a potential diagnostic tool. A 2021 study identified miR-139-5p, miR-151a-5p, and miR-186-5p as predictive markers for the subsequent development of rejection after heart transplantation (153).

T cell-derived exosomal miR-142-3p is elevated during cardiac allograft rejection, contributing to increased vascular permeability by downregulating the expression of the endothelial Rab11 family of interacting proteins 2 (RAB11FIP2) (154).

miR-146a and miR-155 are involved in the regulation of immune response and rejection mechanisms. Deletion of miR-146a in Tregs exerts tissue-protective effects and transiently prolongs cardiac survival in transplanted mice (155). miR-155 serves as a regulator of allograft rejection by affecting T cell proliferation and macrophage function (142, 156, 157).

Inhibition of miR-155 has shown promising results in suppressing macrophage maturation, downregulating T cell responses, and inducing graft immune tolerance. Using antagomiR-155 delivered through ultrasound-targeted microbubble destruction technology reduces the degree of ACR and improves allogeneic heart survival (158). Ultrasound-guided microbubble disruption technology, capable of delivering cationic microbubbles with miRNA155 silencers to target tissues, is considered a more desirable immunosuppressive therapy for ACR (159).

While these studies highlight the potential of miRNAs as diagnostic markers and therapeutic targets in heart transplantation, further research is necessary to validate these findings in larger cohorts and to standardize diagnostic approaches, considering the heterogeneity of treatment protocols across transplant centers. Developing miRNA-based interventions holds promise for improving rejection detection and for managing post-transplantation outcomes in heart transplantation.

## 4 Potential associations of TIM proteins with miRNAs

The relationship between miRNAs, specifically miR-155, and the TIM-3 pathway has been extensively studied in the context of various inflammatory and immune responses, including chronic infections and transplantation. However, the direct implications and specific roles of miR-155 and TIM-3 in allograft tolerance and transplantation immunity need to be further elucidated.

miR-155 is a crucial regulator of inflammation and immunity, affecting various immune cell activities such as macrophage polarization, differentiation of T helper cell subsets such as Th17 and Tregs, and cytokine production (160). miR-155 modulates the expression of suppressor of cytokine signaling 1 (SOCS1), a key negative regulator of the JAK–STAT pathway (161). This miRNA can influence macrophage phenotypes, including the M1/M2 balance, and affect the local inflammatory response in certain contexts, such as liver transplantation and hepatic IRI (32, 162, 163).

TIM-3, an inhibitory co-receptor expressed on immune cells, interacts with different ligands such as Gal-9 and plays a role in regulating immune responses (164, 165). Through its interactions, TIM-3 affects T cell polarization, cytokine production, DC maturation, and other immune activities (166, 167). The interplay between TIM-3 and miR-155 has been studied in inflammation and immune regulation, particularly in controlling adaptive and innate immune cell activation. However, direct evidence regarding their roles in allograft tolerance, specifically in transplantation immunity, is yet to be thoroughly investigated. Understanding the specific contributions of miR-155 and TIM-3 in allograft tolerance might offer potential therapeutic avenues for modulating immune responses and improving transplantation outcomes.

The interactions between other miRNAs (miR-142 and miR-330) and members of the TIM family (TIM-1 and TIM-3) have been studied in various contexts, shedding light on their roles in immune regulation, inflammatory responses, and tolerance induction in different physiological settings, including transplantation and maternal-fetal tolerance (23, 26, 59, 168). Studies have shown that miR-142-3p plays a role in modulating TIM-1 transcription, influencing endothelial cell permeability, and reducing systemic inflammatory responses during viral infections (169, 170). It reports that miR-142-3p are upregulated in biopsies from patients with microvascular inflammation typical of Antibody-mediated rejection (ABMR) (171). Elevated miR-142 levels have been observed in patients with cardiac and renal transplant rejection, indicating its potential as a biomarker for monitoring graft rejection. The regulatory function of miR-142 in targeting TGF-Β sensitivity and enhancing Treg development has been linked to promoting cardiac allograft tolerance by targeting *Tgfbr1* (168). Contradictory findings have been reported regarding the effects of miR-142 knockdown in specific cells. While Treg-specific knockdown led to severe autoimmune disease, transient knockdown enhanced Treg survival and improved skin graft survival (172). After *in situ* liver transplantation, TIM-1 blockade not only inhibits macrophage recruitment and infiltration, but also enhances Th2/Treg differentiation and improves IRI (173). TIM-1 signaling, in turn, can maintain and induce baseline levels of Bregs and clear apoptotic cells during transplantation to produce IL-10, which promotes immune tolerance and survival (46). Even TIM-1^+^ Bregs affect Th differentiation, thereby inhibiting Th1/Th17 cells and promoting Th2 cells and Foxp3^+^ Tregs, which are dependent on IL-10 expression (174).

miR-330-5p protects against myocardial IRI and apoptosis by modulating TIM-3 transcription and translation, thereby reducing the expression of the inflammatory mediator NLRP3 (24). In a model of myocardial IRI, downregulation of miR-330 inhibited left ventricular remodeling via the TGF-Β1–Smad3 pathway (175). miR-330–TIM-3 interactions promote macrophage M2 polarization, inhibiting local inflammation and insulin resistance (26). TIM-3 activity in innate immune cells, facilitated by miR-330, contributes to trophoblast invasion and angiogenesis, essential for maintaining maternal-fetal tolerance (176).

## 5 Hypothetical insights from the mechanism process

Although there is no direct evidence in the literature suggesting that miRNA and TIM may play an emerging role in transplantation immunity. However, we seem to be able to propose a plausible hypothesis for such an interaction mechanism through the signaling axis they share.

The miRNA/TIM/TLR signaling axis: In a model of lung transplantation, miR-21 and miR-122 ameliorate graft dysfunction and ischemia-reperfusion injury by negatively regulating the TLR signaling (177). Activation of the TLR signaling pathway alters macrophage miR-21 expression, which influences macrophage polarization status and inflammatory responses (178). The interaction between the two acts as a feedback regulator that modulates the initiation and termination of inflammation, providing a fundamental argument for post-transplant immune regulation (179). Furthermore, in addition to TLRs themselves, miRNAs also regulate TLR-related signaling proteins that regulate related pathways. For example, in Kupffer’s disease, miR-146a/b can act as a negative regulator to control the TLR4 pathway to prevent liver transplant injury by down-regulating IRAK1 and TRAF6 (180). TIM-3 inhibits the production of inflammatory factors associated with the TLR pathway by suppressing NF-ΚB to create an immune-tolerant microenvironment (181). Interestingly, HMGB1 promotes TIM-1Breg cell expansion through TLR2/4 and mitogen-activated protein kinase (MAPK) signaling pathways, providing new evidence for immune tolerance (182). Surprisingly, miRNAs were able to attenuate inflammatory and oxidative responses through the HMGB1/TLR4/NF-ΚB axis (183). Although the relationship between miRNAs and TIM-targeted regulation has long been clear. However, data show that miRNAs bind to mRNAs encoding the 3’-UTR of TIM-3 (36). All these data are sufficient to suggest that the miRNA/TIM/TLR may become a new signaling axis for immune regulation before and after transplantation.

miRNA/TIM/PI3K/AKT signaling axis: The ability of miRNAs to make early prediction and intervention of post-transplantation acute kidney injury through PI3K/AKT signaling pathway was found by prediction (184). miR-21 accelerates wound healing and angiogenesis in grafted skin by activating PI3K/AKT and ERK1/2 signaling (185). Upregulation of miR-221 was able to target PTEN to activate PI3K/AKT to restore contractile function and ameliorate myocardial injury in transplanted myocardium (186). Binding of Gal-9 to Tim-3 can inhibit activation of the PI3K/AKT pathway and enhance the function of Treg cells, thereby attenuating acute GVHD and inducing immune tolerance (187). In the AML model, elevated TIM-3 promotes M2 macrophage polarization, leading to elevated PI3K and AKT levels to accelerate tumor immune escape (188). Through PI3K/AKT signaling, it has long been clear that miRNAs can promote tumor metastasis, immune escape and microenvironmental remodeling (189). MiRNAs have a novel mechanism to balance immune injury and tolerance in viral infection and anti-tumor with respect to TIM signaling capacity in T/NK cells (190). In summary, we believe that induction of immune tolerance and improvement of graft function in the transplant microenvironment are the main themes of this pathway.

TIM/miR/SOCS1 signaling axis: Recent literature suggests that miR-142 and miR-155 exhibit differential expression patterns in the miRNA profiles of kidney transplant samples, with both being upregulated in biopsies from patients exhibiting microvascular inflammation characteristic of rejection (146, 171). Furthermore, it has been demonstrated that miR-155 directly targets SOCS1, thereby promoting immune cell activation and enhancing the immune response (161, 191). Collectively, these findings indicate that modulation of the miR-155/SOCS1 axis may offer novel insights into the mechanisms underlying transplantation immunity. In a similar vein, the miR-142/SOCS1 axis may play a significant role in disease pathogenesis by influencing T cell differentiation and enhancing the secretion of specific cytokines, including IL-6 and IL-8 (192, 193). These effects can adversely impact transplanted organs and elevate the risk of graft rejection. As illustrated in **Table 1**, existing studies have validated the regulatory roles of miR-142 and miR-155 in the modulation of TIM-1 and TIM-3, respectively.

These findings suggest that intricate interactions between miRNAs and members of the TIM family modulate immune responses, regulate inflammatory processes, and influence tissue-specific responses. These interactions can have diverse effects on various immune cells, leading to implications in transplantation tolerance, inflammation modulation, and maternal-fetal immune regulation. Further studies are required to better understand the precise mechanisms and outcomes of miRNA–TIM interactions in transplantation settings and harness their potential for therapeutic interventions aimed at promoting immune tolerance and mitigating transplant rejection.

## 6 Discussion

The field of transplantation medicine has evolved substantially over the years, offering life-saving treatments for individuals with organ failure or tissue damage. Despite these advancements, post-transplantation complications remain a considerable challenge. Issues such as graft rejection, IRI, allograft dysfunction, and infections can jeopardize successful organ transplantation. Enhancing long-term graft function and survival outcomes requires a personalized treatment approach tailored to individual immune responses.

The TIM family of proteins is a focal point of transplantation research. Modulation of the TIM pathways using blocking antibodies or soluble proteins has shown promise in altering immune responses. These approaches aim to tilt the balance toward tolerance by providing co-inhibitory signals to T and B cells or suppressing innate immune cells. However, varying affinities and epitopes of TIM antibodies can lead to different T cell effects, resulting in immune cell dysfunction. Moreover, TIM proteins act as receptors for phosphatidylserine, contributing not only to the regulation of innate immunity but also to the control of adaptive immune responses, adding complexity to their roles in transplantation.

miRNAs are key regulators of gene expression and have shown promise in transplantation immunology. Analysis of circulating and tissue-specific miRNAs has suggested them as diagnostic and prognostic biomarkers, offering insights into efficacy and predicting transplantation outcomes. These miRNAs hold the potential as the specific markers for assessing immune responses and status of transplanted organs.

Importantly, a reciprocal regulatory relationship exists between the TIM proteins and miRNAs. TIM proteins can regulate miRNAs through various mechanisms; conversely, miRNAs can influence the expression of TIM proteins. This intricate interplay has been observed in various contexts, including tumorigenesis, viral infections, and metabolic disorders, such as insulin resistance in diabetes mellitus. Exploring and understanding this reciprocal regulation in the context of transplant immune tolerance can offer new avenues for clinical studies and potential therapeutic interventions.

In the realm of future transplantation research in miRNA and TIM, several promising avenues beckon our exploration. Initially, we should focus on the study of specific miRNAs, such as miR-21, miR-155, and miR-133a-5p. Utilizing databases and software like miRWalk and TargetScan, we can predict potential binding sites for these miRNAs. Concurrently, in the context of transplantation, it’s imperative to collect plasma, urine, or tissue samples from patients before and after transplantation or drug administration. These samples can undergo miRNA sequencing, followed by screening and validation of differentially expressed genes. To investigate downstream signaling molecule alterations, protein microarrays can be employed to identify differential proteins, which can then be verified using luciferase reporter genes for miRNA binding to the 3’UTR of genes.

Furthermore, the expression patterns of miRNAs may vary between different transplanted organs, indicating tissue-specific regulatory mechanisms. Hence, we should prioritize the study of post-transplantation immunomodulatory capacity on miRNA. This includes the regulation of immune cell function and response strength in adaptive immunity (T/B cells) and innate immunity (NK and macrophages). In terms of signaling pathways, our focus should be on influencing cell differentiation/activation/effector function, integrating transcriptomic, proteomic, and other multi-omics data with experimental validation for comprehensive analysis and screening.

Ultimately, leveraging the regulatory role of miRNAs, it’s crucial to devise novel therapeutic strategies for a safe and effective approach to the transplantation site. Nanoparticle delivery technology can be utilized to transport specific immunomodulatory genes to transplanted tissues, thereby inducing local immunosuppressive cytokine production and fostering immune tolerance. Additionally, considering the fragility of miRNAs, Ultrasound Targeted Microbubbles Destruction offers a non-invasive, targeted gene delivery technique that is safe, efficient, and specific.

In summary, the intersection between TIM proteins and miRNAs represents a promising area for further investigation of transplantation immune tolerance. Understanding the complex interplay between these molecules and their regulatory roles may lead to innovative therapeutic strategies aimed at promoting immune tolerance and improving long-term outcomes in transplant recipients.
